# Supplementary material for: Identification of photoperiod-induced specific miRNAs in the adrenal glands of Sunite sheep (Ovis aries)
Source: Front Vet Sci. 2022 Jul 22;9:888207. doi: 10.3389/fvets.2022.888207 (PMC9354845; doi:10.3389/fvets.2022.888207)
Supplement: Supplementary file 1 [file Data_Sheet_1.ZIP › Supplementary file/miRDeep2 file.zip!/miRDeep2 file/Novel_Structure/SPLP42A1/Novel_588.pdf]

Provisional ID	:	NC_019484.2_32568
Score total	:	2.4
Score for star read(s)	:	-1.3
Score for read counts	:	0
Score for mfe	:	2.1
Score for randfold	:	1.6
Score for cons. seed	:	
Total read count	:	214
Mature read count	:	210
Loop read count	:	0
Star read count	:	4

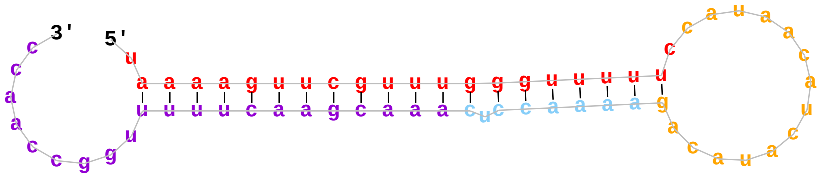

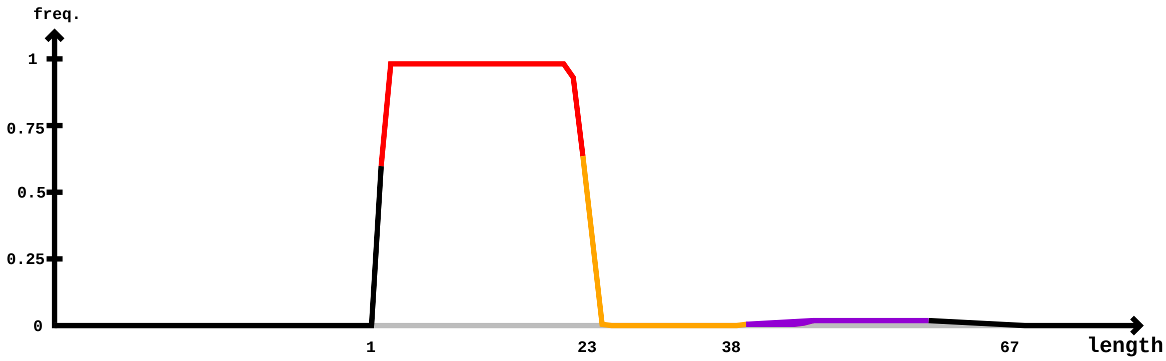


```
                                                 Mature                                        Star
5'- uuaucagacuggccucuagcuauuggguuggcuaaaaguucguuuggguuuuuccauaacaucauacagaaaaccucaaacgaacuuuuuggccaacccaaugcuacaugg  -3'   obs
    uuaucagacuggccucuagcuauuggguuggcuaaaaguucguuuggguuuuuccauaacaucauacagaaaaccucaaacgaacuuuuuggccaacccaaugcuacaugg        exp
    ............((..((((.((((((((((((((((((((((((((((((((...............))))))).))))))))).))))))))))))))))))))...))  reads   mm   sample
    ................................Aaaaaguucguuuggguuuuu..........................................................  62      1    seq
    ................................Aaaaaguucguuuggguuuuuc.........................................................  66      1    seq
    .................................aaaaguucguuuggguuuu...........................................................  10      0    seq
    .................................aaaaguucguuuggguuuC...........................................................  1       1    seq
    .................................aaaaguucguuuggguuuuG..........................................................  1       1    seq
    .................................aaGaguucguuuggguuuuuc.........................................................  2       1    seq
    .................................aaaGguucguuuggguuuuuc.........................................................  1       1    seq
    .................................aaaaguucguuuggguuuuuA.........................................................  3       1    seq
    .................................aaaaguucguuuggguuuuucG........................................................  7       1    seq
    .................................aaaaguucguuuggguuuuucA........................................................  56      1    seq
    .................................aaaaguucguuuggguuuuucAa.......................................................  1       1    seq
    ......................................................................aaaccucaaacgaacuuuuug....................  1       0    seq
    ............................................................................caaacgaacuuuuuggccaacU.............  1       1    seq
    .............................................................................aaacgaacuuuuuggcUaacc.............  1       1    seq
    .............................................................................aaacgaacAuuuuggccaacc.............  1       1    seq
```
